# Patient-centeredness to anticipate and organize an end-of-life project for patients receiving at-home palliative care: a phenomenological study

**DOI:** 10.1186/s12875-017-0602-8

**Published:** 2017-02-23

**Authors:** Agnès Oude Engberink, Mélanie Badin, Philippe Serayet, Sylvain Pavageau, François Lucas, Gérard Bourrel, Joanna Norton, Grégory Ninot, Pierre Senesse

**Affiliations:** 1 Department of General Medicine, University of Montpellier, Montpellier, France; 2 CEPS Platform, University of Montpellier, Montpellier, France; 3 Epsylon EA4556, Dynamic of Human Abilities and Health Behaviors, University of Montpellier, Montpellier, France; 4 Inserm U1061, Montpellier, France; 5 Université de Montpellier, U1061, Montpellier, France; 6 SIRIC Montpellier Cancer, Institut Régional du Cancer de Montpellier (ICM), Montpellier, France; 7 Department of Clinical Nutrition and Gastroenterology and supportive Care, Institut Régional du Cancer de Montpellier (ICM), Montpellier, France; 8 Centre de Médecine Générale, 2 rue IBN Sinai dit AVICENNE, Cabestany, 66330 France

**Keywords:** General practice, Palliative care, Patient-centred approach, Qualitative phenomenological study, Semiopragmatic analysis

## Abstract

**Background:**

The development of end-of-life primary care is a socio-medical and ethical challenge. However, general practitioners (GPs) face many difficulties when initiating appropriate discussion on proactive shared palliative care. Anticipating palliative care is increasingly important given the ageing population and is an aim shared by many countries.

We aimed to examine how French GPs approached and provided at-home palliative care. We inquired about their strategy for delivering care, and the skills and resources they used to devise new care strategies.

**Methods:**

Twenty-one GPs from the South of France recruited by phone according to their various experiences of palliative care agreed to participate. Semi-structured interview transcripts were examined using a phenomenological approach inspired by Grounded theory, and further studied with semiopragmatic analysis.

**Results:**

Offering palliative care was perceived by GPs as a moral obligation. They felt vindicated in a process rooted in the paradigm values of their profession. This study results in two key findings: firstly, their patient-centred approach facilitated the anticipatory discussions of any potential event or intervention, which the GPs openly discussed with patients and their relatives; secondly, this approach contributed to build an “end-of-life project” meeting patients’ wishes and needs. The GPs all shared the idea that the end-of-life process required human presence and recommended that at-home care be coordinated and shared by multi-professional referring teams.

**Conclusions:**

The main tenets of palliative care as provided by GPs are a patient-centred approach in the anticipatory discussion of potential events, personalized follow-up with referring multi-professional teams, and the collaborative design of an end-of-life project meeting the aspirations of the patient and his or her family. Consequently, coordination strategies involving specialized teams, GPs and families should be modelled according to the specificities of each care system.

## Background

According to a 2013 ONFV report (*Observatoire National de la Fin de Vie*, French National End-of-Life Observatory) [1], 25% of French people died at home; professionals lack adequate training. In its recommendations, this report notes that end-of-life conditions should be improved. Regardless of the WHO (World Health Organization) recommendations palliative care is often restricted to a reactive approach and to the relief of physical symptoms in the terminal phase [2].

During the 2015 MASCC (Multinational Association of Supportive Care in Cancer) conference, LH Einhorn reminded attendees that “early palliative care” and “symptoms relief” were two of the five main challenges in oncology [3]. Meeussen [4] revealed that many patients die in places they had not chosen, with often unwanted interventions.

To facilitate identification of patients in need of palliative care several sets of indicators have been developed [2] such as the Supportive and Palliative Care Indicators Tool (SPICT) in Scotland, The Prognostic Indicator Guide (PIG) in England, and the Radboud Indicators for Palliative Care needs (RADPAC) in the Netherlands. In a Dutch RCT [5] GPs in the intervention group were trained in identifying patients in need of palliative care and anticipatory care planning. Next, for each identified patient, they were offered a coaching session with a specialist in palliative care. The control group did not receive any training or coaching. The study did not find any difference between the intervention and control groups in out-of-hour contacts, contacts with their own GP, hospitalizations or place of death. However, the authors conclude that better adapted tools and training programmes may be necessary to show an improvement on identification of patients in need of palliative care and providing adequate care.

In Europe, GPs have difficulties identifying at the right time the need for palliative care, as shown in Belgium [6]. GPs found it difficult to determine the right moment to implement palliative care and to interpret patient aspirations; they reported that they could only help patients in their last year of life.

An Australian systematic review [7] surveyed 66 studies published between 1966 and 2000 relevant to GP provision of palliative care. They corroborate to show that GPs value this area of their work, and patients appreciate their involvement when their physicians are available, make time to listen to them, allow them to express their feelings and make efforts to alleviate their symptoms. In the Netherlands, where GPs have historically been involved in palliative care, a qualitative study [8] of 22 GPs used 3 focus groups to examine their perceptions. The GPs described their palliative care tasks as satisfactory but burdensome. The challenges they faced were categorized according to three levels: 1) personal (knowledge, skills, emotions), 2) relational (communication and collaboration), and 3) organizational (organization of care and compartmentalization in healthcare).

Major barriers to effective home palliative care and end-of-life appear to be lack of medical training and health organization. In two studies surveying 62 medical schools, Sullivan et al. [9, 10] showed that students were critical of their training: they felt it lacked educational expertise and role-models, and that it focused more on cure-driven practices than on care-driven ones. Slort [11] identified the main barriers for GP-patient communication in a systematic review (fifteen qualitative studies and seven quantitative questionnaire studies): lack of availability and GPs’ ambivalence to discuss palliative prognosis. Also, in a recent French study [12] 87 GPs reported on their involvement in the decision-making process for assigning patients to palliative care. Seventy percent of these GPs felt that their involvement in the care strategy was practically nil, and 83% reported that they had not been consulted on decisions regarding the limitation or cessation of therapeutics by hospital staff. In addition, a specialist doctor usually becomes the primary caregiver for patients in their final stage of life.

Taken collectively, the above-mentioned studies stress the importance of structuring organized palliative care in coordination with primary care. They also raise the issue of adequate GP-patient communication training for students and practicing GPs, as well as other relevant skills to develop.

Thus, exploring the lived experience of GPs providing at-home palliative care is a relevant topic in many countries; a better understanding of their approaches and practices would likely allow us to formulate improvements which could impact the quality of the palliative care received by patients.

## Methods

### Aim

We performed a qualitative study to determine how French GPs provide palliative care in at-home settings, what their needs may be, and what skills and resources they mobilize for these interventions.

### Design

We used a phenomenological approach to analyse the semi-structured in-depth interviews of GPs regarding their palliative care lived experience. Subsequently, we carried out a semiopragmatic analysis of the data.

The study’s design met COREQ criteria [13] (COnsolidated criteria for REporting Qualitative research).

### Setting

#### Characteristics of participants

GPs were selected from the *French Regional College of General Practitioners* in the Languedoc-Roussillon region, and contacted by telephone by the investigator (FL). We used purposive sampling to obtain a diversity of GP experiences across various characteristics such as age, sex, work setting (alone or in a team, urban or rural), and years of practice. We applied the principle of data saturation without pre-defining the number of interviews.

#### Data collection

An interview guide (Table 1) including phenomenological questioning focused on lived experience was developed by the methodology team. Two qualitative methodology experts (AOE, MD; GB, MD) verified the appropriateness, as well as the intelligibility, of the questions in two initial test sessions administered by the investigator. Follow-up prompts were designed to lead participants to recount their personal experience.

**Table 1:** Semi-structured in-depth interview guide (phenomenological approach)

What does the palliative care process mean to you, and where does it fit within the practice of general medicine? Prompt: How do you feel about Palliative Care in general medicine?
How do you feel about providing at-home palliative care to patients? Prompt: How do you feel about providing care to end-of-life patients?
Think about one of your palliative care experiences (the most recent one or one which made a mark – positive or negative – on you). Tell me about it. Prompt: How did it go? Do you remember what your thoughts were then? What your feelings were? What you did? How did you feel about it? Why? Do you recall another experience, a “different” one?
What challenges do you face when providing palliative care? Prompt: What type of skills would you like to improve to be better prepared?
In your opinion, can at-home palliative care be implemented, and what are the requirements for it to be provided under good conditions?

The semi-structured interviews were carried out in the workplaces of participants. The investigator introduced himself as a general medicine intern working on a thesis on palliative care. The interviews were recorded with a Philips digital recorder. The sound quality was sufficient to produce audible and understandable voice files. The interviewer made sure to create an atmosphere of confidence such that answers would be spontaneous and truthful. The respondents were informed that their responses would remain confidential and anonymized as they would not be aggregated with their personal identification details. They were made aware of the possibility to stop the interview at any time without any reason. The recordings were transcribed completely and faithfully. We did not plan to collect non-verbal data.

#### Ethical considerations

The study was conducted in accordance with all French regulations. Participants provided written consent for the publication. Approval from the French Ethical Research Committee (Comité de Protection des Personnes, CPP) was sought but deemed unnecessary because of the non-pharmaceutical bio-medical nature of the research [14].

#### Data analysis

Phenomenology is a descriptive method for categorizing lived experience recorded in interview transcripts. The various steps of this analysis were performed according to a method similar to that of grounded theory [15, 16] and completed by a semiopragmatic data interpretation procedure inspired by C. S. Peirce [17, 18]. In this method, the analyst takes into account all the semiotic elements of a text, including linguistic clues, as well as contextual ones. Categories emerge by constant comparison. In our study, semiopragmatic analysis allowed the logical ordering of these empirical categories according to Peirce’s classes of signs. Typically, as a result of this ordering, the conceptually densest category (i.e., of highest level in the hierarchy of signs) commanded the meaning of the phenomenon at play.

The steps of our analytical process are described in Table 2.

**Table 2:** Semiopragmatic analysis steps

Accurate transcription of the recordings (French: verbatims).
Identifying the most relevant elements of contextual anchoring.
General reading, followed by targeted reading.
Dividing text in meaning sections.
Identifying all indexical, textual and contextual elements informing the construction of a category (categorizing by continuous comparison).
Semiopragmatic characterization of emergent categories according to their semiotic level.
Arranging those categories according to their logical inter-relationships.
Constructing the meaning of an emerging phenomenon via a general proposition.

#### Reliability criteria

This work hypothesized an internal methodological consistency between the researched object (lived experience), the phenomenological approach to data collection, and the data analysis favouring a logic of emergence.

The investigator received preliminary training on phenomenological reformulation (prompts) in order to carry out in-depth interviews. He reported his involvement after each interview. Investigator triangulation was achieved by compiling the analyses of the two qualitative research experts and the trained investigator. The interviews were stopped upon reaching data saturation, without the need to add more participants.

Semiopragmatic analysis uses a priori sign classes and adds precision to the logical constructs of a studied phenomenon by limiting investigator-related interpretation bias in the final summary.

## Results

### Participant characteristics (Table 3)

**Table 3:** Characteristics of the 15 GPs respondents

GP	Sexe	Age ranges	location	Practice	Years of Practice	Duration (minutes)
P1	F	30–40	Semi rural	Group (2 GPs)	5	30
P2	F	30–40	Semi rural	Group (2 GPs)	4	49
P3	M	50–60	Rural	Solo	24	48
P4	F	50–60	Rural	Group (2 GPs)	24	52
P5	F	50–60	Urban	Group (3 GPs)	22	33
P6^a^	F	40–50	Urban	Group (3 GPs)	9	28
P7	F	40–50	Urban	Group (3 GPs)	14	46
P8	M	60–70	Urban	Solo	30	37
P9	F	40–50	Semi rural	Solo	17	35
P10	M	60–70	Rural	Solo	33	32
P11	F	30–40	Urban	Group (3 GPs)	3	37
P12	F	30–40	Semi rural	Group (4 GPs)	1,5	34
P13	F	30–40	Semi rural	Solo	6	28
P14	M	50–60	Urban	Group (5 GPs)	29	64
P15	F	30–40	Urban	Group (3 GPs)	9	58

^a^had a palliative care training

Of the 30 GPs contacted, 21 agreed to participate in the study. The interviews were carried out between April 1, 2013 and February 28, 2014. Saturation was achieved after 15 interviews, since no new pertinent information was gathered thereafter. Six GPs were not interviewed.

Transcription and punctuation were completed by the investigator, who played the audio files multiple times. The transcripts were numbered from P1 to P15. Triangulation was achieved with the investigator and two semiopragmatic analysis experts. The first two transcripts were analysed jointly; the other records were rigorously compared in vivo until an agreement was reached on conceptual categories.

### Emergent categories

We identified four emergent categories. They are presented here in general present-tense propositions (phenomenological statements) comprising all meaningful elements.

### Palliative care represents another dimension of care: the transition from a disease-centred curative paradigm to a patient-centred multi-dimensional support and end-of-life quality paradigm

For most GPs, the palliative phase corresponded to the end of the curative pharmacological approach. *“You forget the curing part.”* P1,2,3,4,7,13.*. “We’re there to provide care to people, not to diseases.”* P3*.* All of them implemented palliative steps towards a comfortable and qualitative end-of-life for their patients. For most GPs, palliative care was provided “*within a different care dimension*”(P6): It was empathetic, multidimensional, and based on patient’s preferences; it took into account the entire spectrum of suffering as well as existential dimensions. *“What remains is psychological management, rather; comfort, quality of life.”* P1,2, 3, 6, 9, 11. *“And then, maybe, there can also be a spiritual dimension, with religious beliefs, and the question of the meaning of life and of what it is to exist.”* P4, P12, P13, P15. *“Trying to listen to them, it’s important to be available”* P3*. “Since we don’t have much else to offer, we give time.”* P1

### GPs’ patient-centered approach combines duty as a human being with professional and personal values

GPs found that palliative care made them face their own human condition and its limits. *“We’re just Men… and death is part of life.”* P3. Emotionally, several GPs suffered from this confrontation with end-of-life issues affecting patients to whom they were attached. *“I have a real tough time with it, because I get very close to them, and when they start going, it’s very difficult for me…that’s that.”* P10, P12*.* P8 and P15 saw end-of-life caregiving as a moral duty.

*“I’ve never abandoned any of my patients in any such situations. Er… I feel that it’s part of my job, of my duty as a Man”* P8.

The GPs’ palliative care process integrated the values of their profession and their own values. P2 deemed his involvement “*natural*” and *“authentic*”. P3, P7 and P9 talked about “*birth-to-death care*” as a tenet of general medicine. GPs found they were “*in their legitimate place*” when providing palliative care. P3 thought that “*the job is fabulous when you give yourself the means for it*”. P5 and P8 insisted on the need for drug therapy training, and regretted the lack of resources. Others had received complementary training “*relaxation and psychosomatics training*”P9. Only P8 admitted to being “ignorant in palliative care” and relieved by end-of-life hospital care.

The GPs stated that they also tapped into their personal experiences, or in that of their friends and families, to develop palliative care approaches.

*“I think that this type of caregiving can be provided from a personal experience perspective… I mean, the wisdom of […] knowing yourself.”* P7, P3.

### Discussing and anticipating potential events allows GPs to collaboratively devise “end-of-life projects” with their patients

The GPs reported that this process was initiated by an early discussion (upon diagnosis) to anticipate potential intercurrent events and patient wishes. *“For me, anticipating is key, for everything.”* P9*.* Anticipating potential events allows the creation of space for discussion where the patient may verbalize his or her lived experience. *“I try to discuss things with them on the basis of their emotions, about how things might evolve, how they feel about it…”* P9, P15.

For P4, “*anticipating means leaving options open for patients, so they may change their mind at any time*.” Though the GPs did not point to a “perfect moment” at which this discussion may be started, they reported seizing opportunities created by intercurrent events, such as when patients left the hospital (P 2). Anticipating also means designing an “end-of-life project” with patients and families, which helps confirm and validate their status as a living person with “time left to live”, allowing them to exercise free will, up to the choice of place of death. *“And you’re still within life, there is time left to live, still… there’re so many things to do, so much stuff to take care of”* (P5). P1, P7 and P8 stressed that “*the decision-making process must be shared with the patient*.”

### Organizing human presence around the patient by sharing the caregiving amongst a multi-disciplinary team

The physicians sought support from referring hospital doctors, existing networks, and French home care system options (UMSP, Unité Mobile de Soins Palliatifs). *“It feels comfortable to share the caregiving with others.”* P2

Depending on location, GPs have to set up an informal operational network dedicated to patients. *“The first thing is a solid inpatient-outpatient network; generally, I try to surround myself and have relationships with surgeons and oncologists whom I trust.”* P14.

This takes time, and requires significant personal resources. Furthermore, they stressed the need to share the caregiving (with unit, at-home hospital doctors, for example), to develop at-home nursing care with social workers, and to organize informal discussions with peers and nurses. *“Bedside meetings with everyone, caregivers and helpers.”* P7.

All GPs shared the idea that the end-of-life requires human presence, and that it was their mission to organize it around the patient. *“You can’t do anything without the entourage, whether it’s relatives or neighbors.”* P4, P9. Such coordination requires that the physician remain a permanent link available by telephone (P2, P7, P14). *“At any rate, even end-of-life patients have my mobile phone number, or nurses with whom I’ve worked.”* P1.

**Fig. 1:**
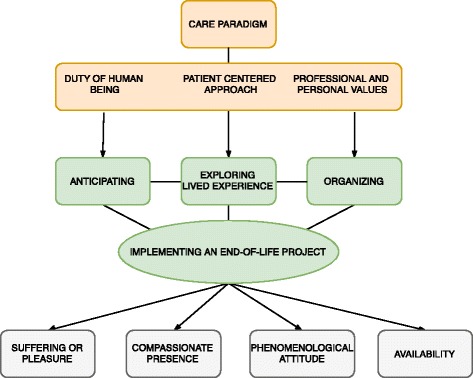
GP’s patient centered approach model in at-home palliative care

Comprehensive statement according to semiopragmatic analysis (Fig. 1) : the GP’s patient centered approach combining duty as a human being, professional and personal values is the core category.

Discussing, anticipating and organizing are the operations of the Patient-Centered Approach in practice, implementing an end-of-life project. These operations mobilize suffering or pleasure, compassionate presence, phenomenological attitude and availability.

## Discussion

Our study showed that, when GPs provided palliative care, they moved from a disease-centred curative paradigm to a patient-centred multi-dimensional paradigm. To them, palliative care was first and foremost their duty as a human being. The experiences reported by the participants showed they felt in their “rightful place” in the palliative care process, tapping into the values of their profession based on GP-patient communication while listening to patients, spending time with them, making time to answer their questions, using their knowledge of the patient’s medical and personal background to do so. In spite of their skillsets (only one GP stated that he felt “incompetent”), they expressed the need for additional support from referring specialists or dedicated infrastructures (networks and socio-medical systems). Moreover, they estimated that, without support from specialists, natural and other care providers, the implementation of the at-home palliative care process was difficult.

For these reasons, and in accordance with results published by Bruera et al. [19], an Integrated Care Model, in which physicians routinely refer patients to palliative care, could help GPs and ensure patients receive comprehensive and integrated care. Most interestingly, in our study, at the core of the GP process is the patient-centred approach which allowed them to anticipate discussion with the patient and their family – of all potential events associated with the disease, its evolution and its psychosocial impact as strongly as recommended [3, 20, 21]. Contrary to the conclusions of Beernaert’s study [5], the GPs in our sample deemed these discussions necessary, emphasizing the importance of their being held early, clearly and truthfully with patients and families, in order to explore their feelings and aspirations from diagnosis to treatment cessation, and even to place of death. Moreover, our study focused on the necessity to include GPs in these new programs. For example, in our study, the GPs could not define the most adequate moment for these discussions to be prompted and held; in practice they tried to seize the most suitable time, such as when patients left the hospital, or when asked a question from the family.

Our study showed that anticipating potential events allowed GPs to devise end-of-life projects which met the aspirations of patients and their families. For these physicians, respecting patient choices was critical to designing a suitable end-of-life project. Their underlying objective was to ensure the highest quality of life possible during the “time their patients had left to live”. Our phenomenological analysis of the lived experiences GPs reported led us to identify the 3 categories of Slort et al.’s ACA training model (Availability, Current issues, Anticipation) [11, 22] as well as the criteria associated with these categories, such as GP availability (listening to patients, spending time with them, contacting them by phone, the multi-dimensional management of issues, and the anticipation of events (end-of-life project, respecting choices). Though GPs found this model valuable, a study comparing an ACA-trained group of GPs and a control group using the Roter Interaction Analysis System (RIAS) did not find any significant difference in palliative care management efficacy [23]. Even though our purposive-selected GPs had not received specialized palliative care training (except one), they were able to muster and implement a patient-centred professional approach.

For these reasons, our study allows us to hypothesise that patient-centred approach training could improve the quality of communication with patients about end-of-life issues, particularly developing lived experience interview skills using phenomenological attitudes [24].

Beyond applying the many available disease-specific indicators, this person-centred approach could be a useful skill to identify patients in need of palliative care. Similarly to the Thoonsen study design [5], it would be interesting to conduct a RCT with GPs in the intervention group trained to develop a phenomenological patient-centred approach.

### Strengths and limitations

Our approach to this work allowed us to meet our objective of a better understanding of how French GPs provide palliative care in at-home end-of-life settings, what their needs may be, and what skills and resources they mobilize for these interventions. The strength of our study lies in our choice to use phenomenological analysis, which enabled the emergence of unexpected categories. Also, we chose to further examine these categories with semiopragmatic analysis in order to highlight the most relevant ones: the patient-centred approach paradigm which enables anticipation— and collaborative discussion — of potential events to facilitate the design and implementation of an end-of-life project which meets the aspirations of the palliative care patient. Our study did suffer from a minor limitation. In spite of having received training on comprehensive interviews, our investigator initially lacked the experience to carry out phenomenological interviews, which should include timely prompts (the key to sound data collection). However, the investigator’s technique quickly improved under the supervision of the experts. Nonverbal data was not collected. Triangulation was properly achieved with the investigator and two transcript analysis experts. To our knowledge, no other such study using semiopragmatic analysis has been performed; the fact that the categories were then organized hierarchically and logically — as planned — did assign meaning to the phenomena studied while limiting investigator bias.

### Practical consequences and perspectives

The findings from this study highlight the need for specific palliative training for GPs and for setting up palliative care coordinating teams; also, suggestions are made regarding strategies to improve GPs’ palliative care practices.

- In addition to the development of palliative care identification indicators, specific training programs based on an interview centered on a patient-lived experience for dealing with human suffering and the caregiving relationship (two central issues in palliative care) should be offered to GP trainees so they may hone relationship, communication and emotional skills. This is essential given the central role expected of GPs as experts in discussing end-of-life questions.
- Anticipating and eliciting all possible events is essential and concerns all the dimensions of care (physical, psychosocial, spiritual and emotional). Discussions must be held early at home between patients, their families and the physician (the “family conference*”* in Bruera’s model [25]), and multi-disciplinary evaluations must be conducted regularly.
- A “shared care” process, developed jointly with the family, and other primary and palliative referring care providers, should be validated and coordinated institutionally. Today, in France, such coordination remains informal and the workload proves to be too heavy for a single GP providing at-home care. The coordination of primary care and hospital care services could be centralized within organizations dedicated to supportive care and possessing the entire array of necessary resources (physicians, nurses, physiotherapists, psychologists, nutritionists, spiritual care, and social workers). An approach such as a coordinated comprehensive care program, would meet the expectations of GPs and correspond to what they attempt to achieve informally.
- Future perspectives are to carry out additional research and experiments on the development of supportive care and on the coordination between primary care and hospital care.

## Conclusion

In summary, our study showed that, despite a relative lack of specialized specific end-of-life training, the GPs in our sample devised a patient-centred approach to palliative care based on their professional values. This process allowed them to anticipate all potential events and discuss them with patients and families. These discussions generated opportunities for GPs to design personalized palliative care projects for the remaining time left to live hand in hand with their patients. Nevertheless, GPs cannot be expected to individually provide comprehensive care. The development and organization of coordinating structures, whether institutional or within primary care facilities and networks, has become critical to provide coordinated and diversified care to patients from a multi-disciplinary team, integrating and complementing the activities of GPs.

## Abbreviations

ACA: Availability current issues anticipation
COREQ: COnsolidated criteria for reporting qualitative research
CPP: Comité de protection des personnes
GP: General practice or general practitioner
MASCC: Multinational association of supportive care in cancer
PIG: Prognostic indicator guide
RADPAC: Radboud indicators for palliative care needs
RCT: Randomised control trial
RIAS: Roter interaction analysis system
SPICT: Supportive and palliative care indicators tool
WHO: World health organization
